# Epidemiology, clinical treatment and outcomes, susceptibility patterns and genotypic analysis of 214 *Nocardia* strains from multiple centers in Henan Province

**DOI:** 10.3389/fcimb.2026.1728269

**Published:** 2026-02-13

**Authors:** Xiaogai Li, Cailin Liu, Yinyin Hu, Hui Xu, Haijun Li, Jingjing Sun, Xiangyang Chen, Yujuan Meng, Nan Zhang, Gongchang Li, Xiuping Lei, Limin Guo, Juhua Chen, Wanhai Wang

**Affiliations:** 1 Department of Clinical Laboratory, The First Affiliated Hospital of Zhengzhou University, Zhengzhou, Henan, China; 2 Key Clinical Laboratory of Henan Province, Zhengzhou, Henan, China; 3 Department of Clinical laboratory, Nanyang Central Hospital, Nanyang, Henan, China; 4 Department of Clinical laboratory, The Fifth Clinical Medical College of Henan University of Chinese Medicine (Zhengzhou People’s Hospital), Zhengzhou, Henan, China; 5 Department of Clinical laboratory, Luoyang Central Hospital, Luoyang, Henan, China; 6 Department of Clinical laboratory, The Fifteenth People’s Hospital of Zhengzhou, Zhengzhou, Henan, China; 7 Department of Clinical laboratory, The Second Affiliated Hospital of Zhengzhou University, Zhengzhou, Henan, China; 8 Department of Clinical laboratory, Xiangcheng City Traditional Chinese Medicine Hospital, Zhoukou, Henan, China; 9 Department of Clinical laboratory, Xinxiang Central Hospital, Xinxiang, Henan, China; 10 Department of Clinical laboratory, Xinyang Maternal and Child Health Care Hospital, Xinyang, Henan, China

**Keywords:** antibiotic resistance, epidemiology, genotyping, *Nocardia*, outcomes, whole genome sequencing

## Abstract

**Objectives:**

This study aimed to investigate the epidemiology, clinical treatment and outcomes, antimicrobial resistance profiles and genotypic analysis of 214 *Nocardia* strains collected from 9 hospitals in Henan Province spanning 9 years.

**Methods:**

Through retrospective analysis of hospitalized patients with nocardiosis, the epidemiological characteristics of 214 *Nocardia* strains were elucidated. These isolates were identified and subjected to the broth microdilution method for the antimicrobial susceptibility profiles, and the resistance and virulence genes were determined using whole-genome sequencing (WGS).

**Results:**

Of all strains, 74.8% were collected from lower respiratory tract specimens, and *N. cyriacigeorgica* was the most commonly isolated species (28%), followed by *N. farcinica* (24.8%), *N. abscessus* (7.9%), *N. amamiensis* (7.9%), *N. otitidiscaviarum* (7.5%). 93.9% were obtained from in-province patients, and Nanyang City (28.0%) was with the highest isolation rate in Henan Province. Simultaneously, all of the strains were susceptible to linezolid (LZD), and 99.1% susceptible to trimethoprim-sulfamethoxazole (TMP-SMX). The antibiotic resistance profiles of other antibiotics varied tremendously among different *Nocardia* species. Of all the patients, 108 (50.7%) received TMP-SMX monotherapy or multidrug regimen; moreover, 182 (85.5%) patients recovered after treatment. Notably, 12 antibiotic resistance genes and 11 virulence genes were identified, implicating the complexity of resistance and pathogenicity mechanisms. Meanwhile, the MDR rates for *Nocardia* species ranged from 68.8% in *N. otitidiscaviarum* to 17.7% in *N. amamiensis.* No strains exhibited the XDR and PDR phenotypes.

**Conclusion:**

This study provides a comprehensive evaluation of the epidemiology, phenotypic and genotypic profiles, and clinical treatment of *Nocardia* species in Henan, China. TMP-SMX and LZD can be used respectively for the clinical routine and critical treatment of nocardiosis. Particular emphasis is placed on the fact that antibiotic resistance and pathogenicity are species - specific, therefore, the AST of *Nocardia* isolates should be conducted and standardized, and attempts should be made to monitor its resistance molecular mechanisms.

## Introduction

The genus *Nocardia* includes ubiquitous environmental saprophytes and the most frequently isolated aerobic actinomycete human pathogen responsible for localized or disseminated infection (Wang et al., 2022; Wang et al., 2023). Infections caused by the *Nocardia* species are referred to as nocardiosis. It is a rare but life-threatening infection caused by the aerobic, gram-positive, and weakly acid-fast bacteria, moreover, is commonly seen in the lower respiratory tract, skin and soft tissues, and central nervous system (CNS) in both immunocompromised and immunocompetent individuals (Yang J. et al., 2023). The genus *Nocardia* usually infects hosts through inhalation of the organism and traumatic inoculation into the skin, especially in patients who have had contact with soil or plant materials. Conditions compromising chronic lung disease, including chronic bronchitis, bronchiectasis, bronchial asthma, chronic obstructive disease, pulmonary tuberculosis, etc., chronic kidney disease, and chronic liver disease increase vulnerability. While systemic immunodeficiency, such as diabetes mellitus, Human Immunodeficiency Virus (HIV), systemic corticosteroids, or chemotherapy, further increases the risk of dissemination (Rathish and Zito, 2025). Specifically, hematogenous dissemination is a vital factor that notably increases mortality rates, with amounting to 85% in immunocompromised individuals (Di et al., 2025; Yang et al., 2025).

The timely diagnosis is essential and relies on a high index of suspicion, aided by gram staining, modified acid-fast staining, prolonged culture, and molecular biological techniques such as matrix-assisted laser desorption/ionization time-of-flight mass spectrometry (MALDI-TOF MS), polymerase chain reaction or gene sequencing technology. Management of initial therapy includes TMP-SMX as topical and first-line therapy, and LZD considered for use in combination therapy for moderate-to-severe or disseminated nocardiosis (Davidson et al., 2020; Wang et al., 2025). Delayed treatment of *Nocardia* infections may produce disseminated infections and multi-organ involvement as systemic infections, and may even cause severe complications such as brain abscesses and meningitis. The prognosis depends on the extent of involvement in the patient and the timeliness of intervention.

Ongoing related studies are constantly updating the taxonomy of the genus *Nocardia*. It’s reported that over 200 *Nocardia* species have been documented (Di et al., 2025), and about 54 of them have been clearly associated with human infections (Hamdi et al., 2020), and this number may be updated. Clinical data show that the main species causing human *Nocardia* infections include the *N. nova* complex, *N. abscessus* complex, *N. otitidiscaviarum*, *N. farcinica*, *N. cyriacigeorgica* and *N. brasiliensis*. The distribution of *Nocardia* species varies geographically, and there are differences in drug susceptibility, carriage of resistance or virulence genes, clinical manifestations, and treatment regimens among different *Nocardia* species, highlighting a significant gap in both recognition and antimicrobial management of clinical nocardiosis. Fortunately, these differences are increasingly recognized and diagnosed by clinical medical personnel.

In the study, by analyzing the species distribution, the clinical characteristics, treatment and outcomes, the drug susceptibility phenotypes and genotypic characteristics, it is hoped to require a deeper understanding of the species diversity, biological properties and virulence mechanisms of *Nocardia* isolates in Henan Province and provide a reference for the diagnosis and treatment decision-making of *Nocardia* infections. What’s more, continuous AST surveillance is essential for characterizing antimicrobial resistance profiles across different regions of our country and for detecting novel resistance traits.

## Materials and methods

### Ethical approval and consent to participate

This study was approved by the Research and Clinical Trial Ethics Committee of the First Affiliated Hospital of Zhengzhou University (2024-KY-2063). Meanwhile, the study has been registered on National Medical Research Registration and Filing Information System (https://www.medicalresearch.org.cn/), record number:MR-41-24-055644. Our study adhered to the Declaration of Helsinki. Since the study was centered around the clinical epidemiological data, phenotype and gene analysis of *Nocardia* isolates, and did not involve human participants and strains were collected as part of the routine clinical management of patients. Therefore, the informed consent was not sought, and an informed consent waiver was approved by the Research and Clinical Trial Ethics Committee of the First Affiliated Hospital of Zhengzhou University.

### *Nocardia* infection cases

The study included 213 cases of *Nocardia* infection that presented from August 2016 to July 2025 at 9 hospitals in Henan Province, China. *Nocardia* infection was defined as at least two positive sputum cultures or one positive culture from transbronchial or lung biopsy, bronchial lavage fluid, blood, other sterile body fluids, skin and soft tissue infections, or tissue specimens, along with clinical signs and/or radiological evidence of organ involvement (lung, skin and soft tissue, brain and peritoneum) (Yang J. et al., 2023).

### Clinical characteristics, treatment, and outcomes

The clinical characteristics of all patients were based on their medical records. Clinical treatment was based on the antibiotic regimens used during hospitalization, and the outcomes were categorized as follows: recovered (clinical symptoms resolved but radiological changes persisted), and failure (no clinical improvement or death following treatment). Information on treatment and outcomes was derived from the medical records.

### Collection and identification of strains

The nonrepetitive *Nocardia* strains collected from each hospital were sent for further examination to the First Affiliated Hospital of Zhengzhou University (Zhengzhou, China). Isolates of *Nocardia* strains were preserved in brain heart infusion broth supplemented with 25% glycerol at -80°C for subsequent studies. The isolation of bacteria and creation of bacterial culture were performed as detailed previously (Han et al., 2024). Species identification was performed mainly using formic acid extraction method of MALDI-TOF MS (VITEK^®^-MS, IVD database, version 3.0, updated November 2022), while in those strains which were failed to achieve species-level identification by MALDI-TOF MS, final identification would be used through the 16S rRNA sequencing.

### DNA extraction, 16S rRNA sequencing

The genomic DNA from each unidentified *Nocardia* strain was extracted using a TakaRa MiniBEST Bacteria Genomic DNA Extraction kit [TaKaRa Bioengineering Company Limited, Dalian, China], following the manufacturer’s protocols. The extracted DNA samples were sent to Ruibo Xingke Sequencing Company (Beijing, China) for 16S rRNA sequencing to obtain the final identification of *Nocardia* species. The sequences were compared using the Basic Local Alignment Search Tool algorithm with the database in the National Center for Biotechnology Information GenBank (http://www.ncbi.nlm.nih.gov). Species identification was based on the similarity value of equal to or greater than 99.6% for 16S rRNA sequencing (Yang J. et al., 2023).

### Antibiotic susceptibility testing *in vitro*

The commercial Sensititre RAPMYCO microdilution panels (ThermoFisher Scientific, OH, USA) were used to detect the AST of the *Nocardia* strains, following the manufacturer’s protocols. The minimum inhibitory concentrations (MICs) of the broth microdilution method were determined for the 15 drugs, including trimethoprim-sulfamethoxazole (TMP-SMX), linezolid (LZD), amoxicillin-clavulanate (AMC), ceftriaxone (CRO), cefepime (FEP), cefoxitin (FOX), imipenem (IPM), amikacin (AMK), tobramycin (TOB), clarithromycin (CLR), ciprofoxacin (CIP), moxifloxacin (MFX), minocycline (MNO), doxycycline (DOX) and tigecycline (TGC). The MIC values of *Nocardia* strains were interpreted as susceptible (S), intermediate (I), and resistant (R) according to the breakpoint criteria of the Clinical and Laboratory Standards Institute (CLSI, M24S-Ed2) for *Nocardia* spp (Parrish et al., 2023). Quality control for AST was ensured by employing *Escherichia coli* ATCC 35218, and *Staphylococcus aureus* ATCC 29213 as reference strains.

### Detection of antibiotic resistance and virulence genes

The presence of antimicrobial resistance and virulence genes were analyzed by WGS in those *Nocardia* strains different from the acknowledged resistance patterns. WGS (denovo) was performed on a HiSeq sequencer (Illumina) following the manufacturer’s instructions by Hangzhou Jieyi Biotechnology Company Limited (Zhejiang, China). FASTQ files were independently assembled using a *de novo* SPAdes genome assembler (version 3.13.1).

### Statistical analysis

The geographic distribution of *Nocardia* species was analyzed using Quantum GIS (version 3.40.10), and the epidemiological data and clinical information data were demonstrated using Microsoft Office Excel 2012. The MICs data for each antibiotic were recorded and analyzed using WHONET 5.6 software, and MIC_50_ and MIC_90_ were calculated, defined as the MICs of a given agent that inhibits the grown of 50% and 90% of the isolates, respectively. The data of the acquired resistance and virulence genes were used to analyze the resistance mechanisms of *Nocardia* through the website (https://www.chiplot.online/tvbot.html).

## Results

### Demographic characteristics and geographical distribution

**Figure 1:**
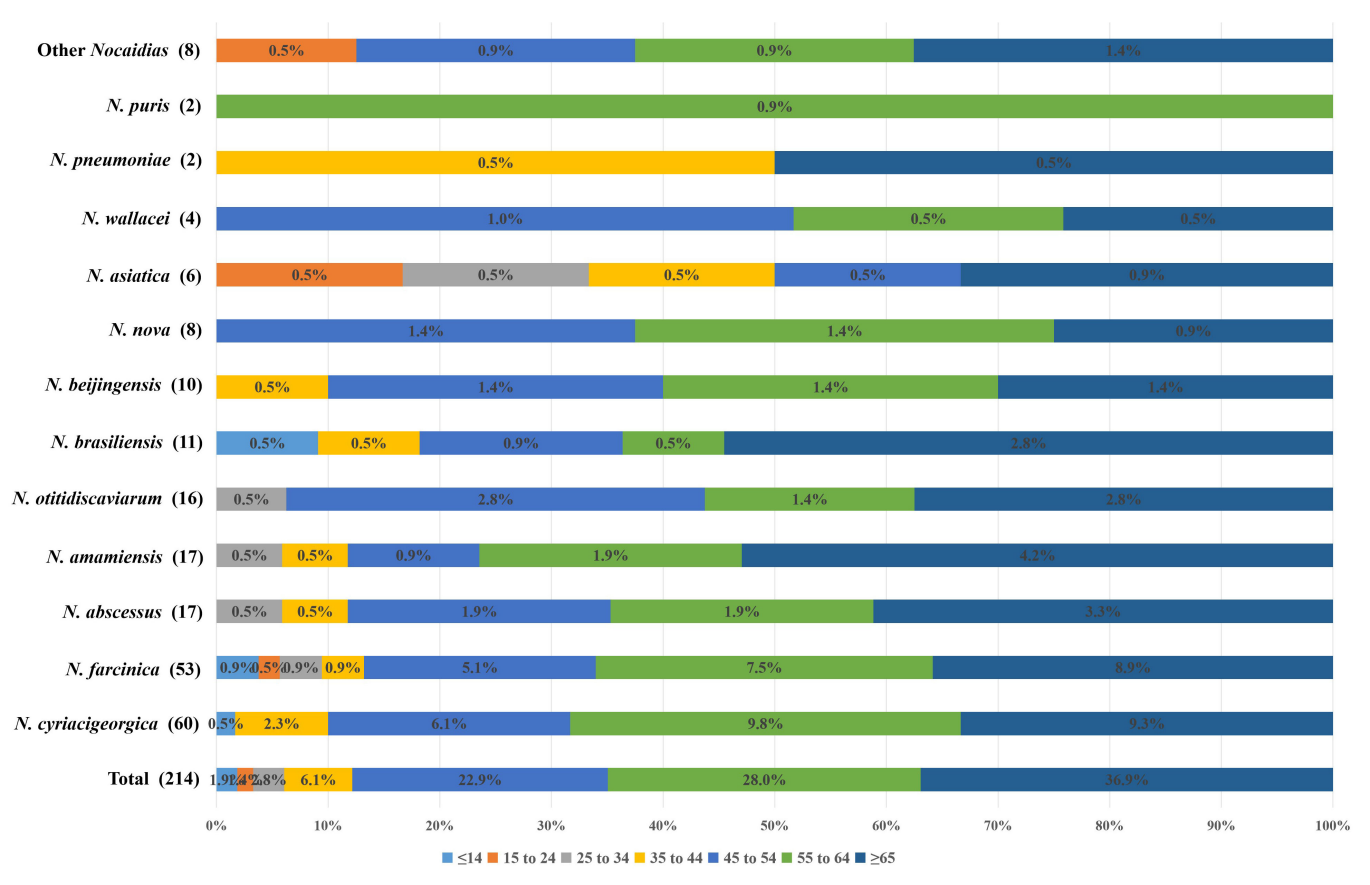
The age distribution (%) of 214 *Nocardia* strains from 2016-2025 in Henan Province.

A total of 213 cases of nocardiosis were involved. Since two different *Nocardia* strains were isolated from the same specimen in 1 case, there were 214 *Nocardia* strains collected. The basic characteristics of 213 nocardiosis cases were summarized in **Table 1**. The median age of all cases was 59 with ranging from 2 to 93 years old, and there were 36.7% (78 of 213) ≥65 years old and only 1.9% (4 of 213) ≤14 years old, and the majority (187, 87.8%) were older than 45 years, as shown in **Table 1** and **Figure 1**. The gender ratio of male/female was approximately 1.3:1 (121/92). Among these cases, the majority of the patients were farmers (116, 54.5%), and the main department involved was respiratory department (114, 53.5%). Please refer to **Table 1** for more details.

**Table 1:** Demographic and clinical characteristics of 214 *Nocardia* strains from 213 patients.

Characteristic	No. of patients (n=213)	Percentage (%)
Age, year [median (IQR)]	59 (52, 69)	
Male, year [median (IQR)]	60 (52, 69)	
Female, year [median (IQR)]	57 (49, 70)	
Age range (years)	2-93	
1 year to14 years	4	1.9%
15 years to 24 years	3	1.4%
25 years to 34 years	6	2.8%
35 years to 44 years	13	6.1%
45 years to 54 years	51	23.0%
55 years to 64 years	58	27.2%
65 years to 74 years	50	23.5%
75 years to 84 years	18	8.5%
85 years to 94 years	10	4.7%
Gender		
Male	121	56.8%
Female	92	43.2%
Occupational distribution		
Farmer	116	54.5%
Urban workers	19	8.9%
Civil servants	2	0.9%
Retirees	23	10.8%
Students	4	1.9%
Others	49	23.0%
Departmental distribution of isolated strains		
Department of Respiratory	114	53.5%
ICU	26	12.2%
Department of Rheumatology & Immunology	11	5.2%
Department of Orthopedics	9	4.2%
Department of Geriatrics	8	3.8%
Department of Infectious Diseases	6	2.8%
Department of Thoracic Surgery	4	1.9%
Department of Neurosurgery	4	1.9%
Department of Nephrology	4	1.9%
Department of Gastroenterology	3	1.4%
Department of Dermatology	3	1.4%
Department of Cardiology	3	1.4%
Department of Pediatrics	2	0.9%
Department of Medical Oncology	2	0.9%
Department of Hematology	2	0.9%
Department of Emergency Medicine	2	0.9%
Department of Cardiac Surgery	2	0.9%
Others	8	3.8%
Infection types and sample sources		
Pulmonary nocardiosis		
Sputum	98	46.0%
Bronchoalveolar lavage fluid	80	37.6%
Lung tissue	6	2.8%
Skin and subcutaneous nocardiosis		
Skin and soft tissue pus	27	12.7%
Central nervous system nocardiosis		
Cerebrospinal fluid	1	0.5%
Brain abscess	4	1.9%
Laboratory-confirmed bloodstream nocardiosis		
Peripheral venous blood	5	2.3%
Others		
Pleural effusion	13	6.1%
Ascitic fluid	3	1.4%
Synovial fluid	2	0.9%
Bile fluid	1	0.5%
Pericardial fluid	1	0.5%
Peripheral intravenous catheter	1	0.5%
Stool	1	0.5%
**Smoking history**	53	24.9%
**Drinking history**	33	15.5%
**Diseases history**	137	64.3%
**Invasive procedure**	98	46.0%
**Empirical anti-tuberculosis treatment**	14	6.6%
**Immunosuppressant use**	39	18.3%
**Chemotherapy history**	13	6.1%
**Steroid use**	81	38.0%
Underlying diseases condition		
Without underlying disease	11	5.2%
Acute or Chronic lung disease	153	71.8%
Hypertension	49	23.0%
Anemia	38	17.8%
Diabetes mellitus	38	17.8%
Acute or Coronary heart disease	37	17.4%
Acute or Chronic liver disease	36	16.9%
Autoimmune disease	35	16.4%
Acute or Chronic kidney disease	34	16.0%
Solid cancer	24	11.3%
Skin and soft tissue infection	24	11.3%
Acute or Chronic brain disease	17	8.0%
Lumbar disc herniation	6	2.8%
Hematological cancer	5	2.4%
Empyema or Pleurisy	5	2.4%
Fracture	4	1.9%
Transplant recipient	2	0.9%

IQR, inter quartile range; ICU, intensive care unit.

Bold text, different categories of clinical data analysis.

The most common specimen sources were recovered from the lower respiratory tract with sputum 46.0%, bronchoalverolar lavage fluid (BLF) 37.6%, and lung tissue 2.8%; 12.7% (27 of 213) were recovered from skin wound, pus, abscess, and soft tissue; 6.1% (13 of 213) were recovered from pleural effusion; 2.8% (6 of 213) were recovered from peripheral venous blood, and 2.3% (5 of 213) were recovered from cerebrospinal fluid and brain abscesses. In our cases, there were two or more different specimen types positive for the same *Nocardia* sp., such as *N. cyriacigeorgica* might isolate from both BLF and sputum in one case, being analyzed as one strain. The sources of 214 *Nocardia* strains were summarized in **Table 1**.

**Figure 2:**
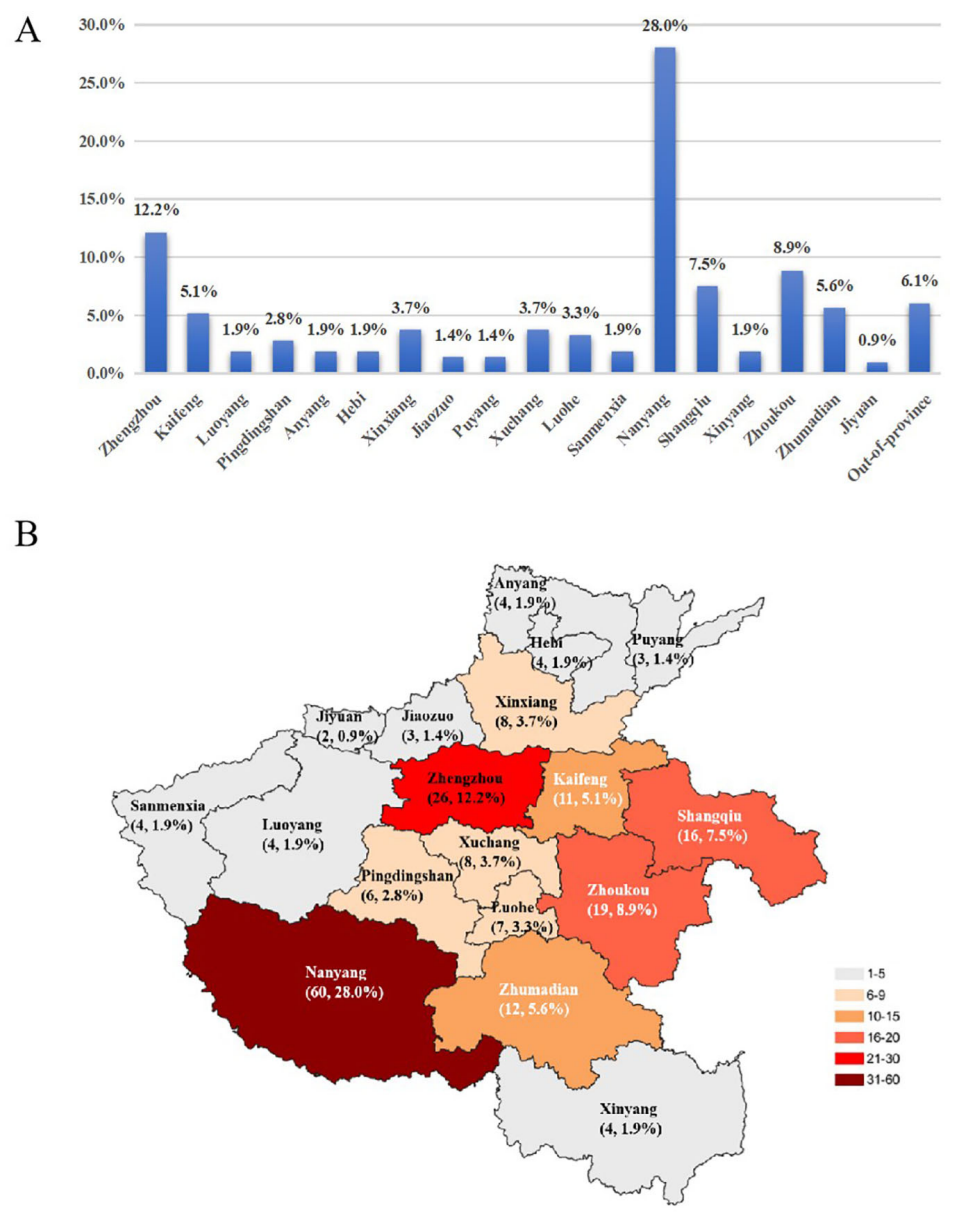
Geographica distribution of 214 *Nocardia* isolates collected from August 2016 to July 2025. **(A)** All 214 *Nocardia* isolates collected in 9 laboratories in Henan Province. **(B)** Geographica distribution of 201 strains collected from patients within Henan Province covering all 18 cities.

As shown in **Figure 2A**, among the 214 strains, 93.9% (201 of 214) were obtained from in-province patients and 6.1% (13 of 214) from out-of-province patients. The geographical distribution of the 201 strains from within the province is shown in **Figure 2B**. Nanyang City (60, 28.0%) was with the highest isolation rate, followed by Zhengzhou (26, 12.2%), Zhoukou (19, 8.9%), Shangqiu (16, 7.5%), Zhumadian (12, 5.6%) with the top 5 of in-province patients. The remaining 13 (6.1%) strains were obtained from patients of Shandong Province (5, 2.3%), Shanxi Province (1, 0.5%), Anhui Province (1, 0.5%), Shanxi Province (2, 0.9%), Heilongjiang Province (1, 0.5%) and the Xinjiang Uyghur Autonomous Region (3, 1.4%), respectively.

### Clinical characteristics

Among the nocardiosis patients, 24.9% (53 of 213) had a history of smoking, 15.5% (33 of 213) had a history of alcohol consumption, 46.0% (98 of 213) had invasive procedure, and about 62.4% (133 of 213) had history of immunosuppressant use, steroid use, and chemotherapy. Of the 213 patients, 5.2% (11 of 213) without underlying disease, 94.8% (202 of 213) had at least one underlying disease. Here, acute and chronic pulmonary diseases had the highest proportion (153, 71.8%), and hypertension (49, 23.0%), anemia (38, 17.8%), diabetes mellitus (38, 17.8%), and acute/coronary heart disease (37, 17.4%) were followed. Among these patients with pulmonary nocardiosis, the most common comorbidities included pulmonary infection (119, 55.9%), bronchiectasis (33, 15.5%), chronic obstructive pulmonary disease (COPD) (19, 8.9%), pulmonary fibrosis (12, 5.6%), and emphysema (11,5.2%). The most common clinical manifestations and signs of nocardiosis were cough (153, 71.8%) and expectoration (148, 69.5%), which were followed by fever (128, 60.1%), and further details shown in **Table 2**.

**Table 2:** Laboratory data, imaging findings, and clinical signs of 213 cases of *Nocardia* infections.

Characteristic	No. of patients (n=213)	Percentage (%)
Chest radiograph		
Unilateral lung involvement	14	6.6%
Bilateral lung involvement	178	83.6%
Pleural involvement	119	55.9%
Patchy consolidation	116	54.5%
Cloud-like or linear shadows	108	50.7%
Nodular shadow	104	48.8%
Cavity shadow	45	21.1%
Air shadow	17	8.0%
Pleural effusion	93	43.7%
Mediastinal or Cervical or Supraclavicular lymphadenopathy	53	24.9%
Intracranial space-occupying lesion	3	1.4%
Clinical manifestations and signs		
Fever	128	60.1%
Cough	153	71.8%
Expectoration	148	69.5%
Hemoptysis	14	6.6%
Chest pain	22	10.3%
Dry or wet rales	76	35.7%
Wheezing	14	6.6%
Altered consciousness	10	4.7%
Meningeal irritation signs	1	0.5%
Subcutaneous abscess	23	10.8%
Laboratory data		
WBC increased	120	56.3%
Increased proportion of NEU (%)	137	64.3%
C- reative protein elevation	154	72.3%
PCT elevation	129	60.6%
IL-6 elevation	63	29.6%
Increased ESR	105	49.3%
Elevated ferritin Levels	42	19.7%
Hypoproteinemia	117	54.9%
Co-infection		
No	77	36.2%
Respiratory viruses	70	32.9%
HBV or HCV or HIV	19	8.9%
MTB	8	3.8%
NTM	2	0.9%
*Legionella pneumophila*	1	0.5%
Other bacteria	75	35.2%
Filamentous fungi	42	19.7%
Candida	28	13.2%
*Pneumocystis jirovecii*	19	8.9%

WBC, white blood cell; NEU%, neutrophil percentage; ESR, erythrocyte sedimentation rate; HBV or HCV or HIV, Hepatitis B Virus or Hepatitis C Virus or Human Immunodeficiency Virus; MTB, *mycobacterium tuberculosis*; NTM, Non-*tuberculous mycobacteria*.

During the diagnostic process for nocardiosis, 205 patients underwent complete imaging. Through CT scans, it was found that unilateral lung involvement accounted for 6.6% (14 of 213), while bilateral lung involvement was as high as 83.6% (178 of 213), followed by pleural involvement for 55.9% (119 of 213), patchy consolidation for 54.5% (116 of 213), respectively. In the laboratory data, 56.3% (120/213) had elevated white blood cell counts, and 64.3% (137/213) had increased neutrophil proportions. Additionally, the rates of elevated inflammatory factors, including CRP (72.3%), ESR (49.3%), PCT (60.6%) and IL-6 (29.6%), also varied. Besides, 54.9% of patients had hypoproteinemia, and 19.7% involved elevated ferritin levels. Among them, 0.5% (1 of 213) were determined to be *Nocardia* colonization, and 63.4% cases (135 of 213) co-infected with other pathogens. These cases included 32.9% (70 of 213) of respiratory virus infection and 3.8% (8 of 213) of MTB infection, as summarized in **Table 2**.

### Distribution of *Nocardia* species

**Figure 3:**
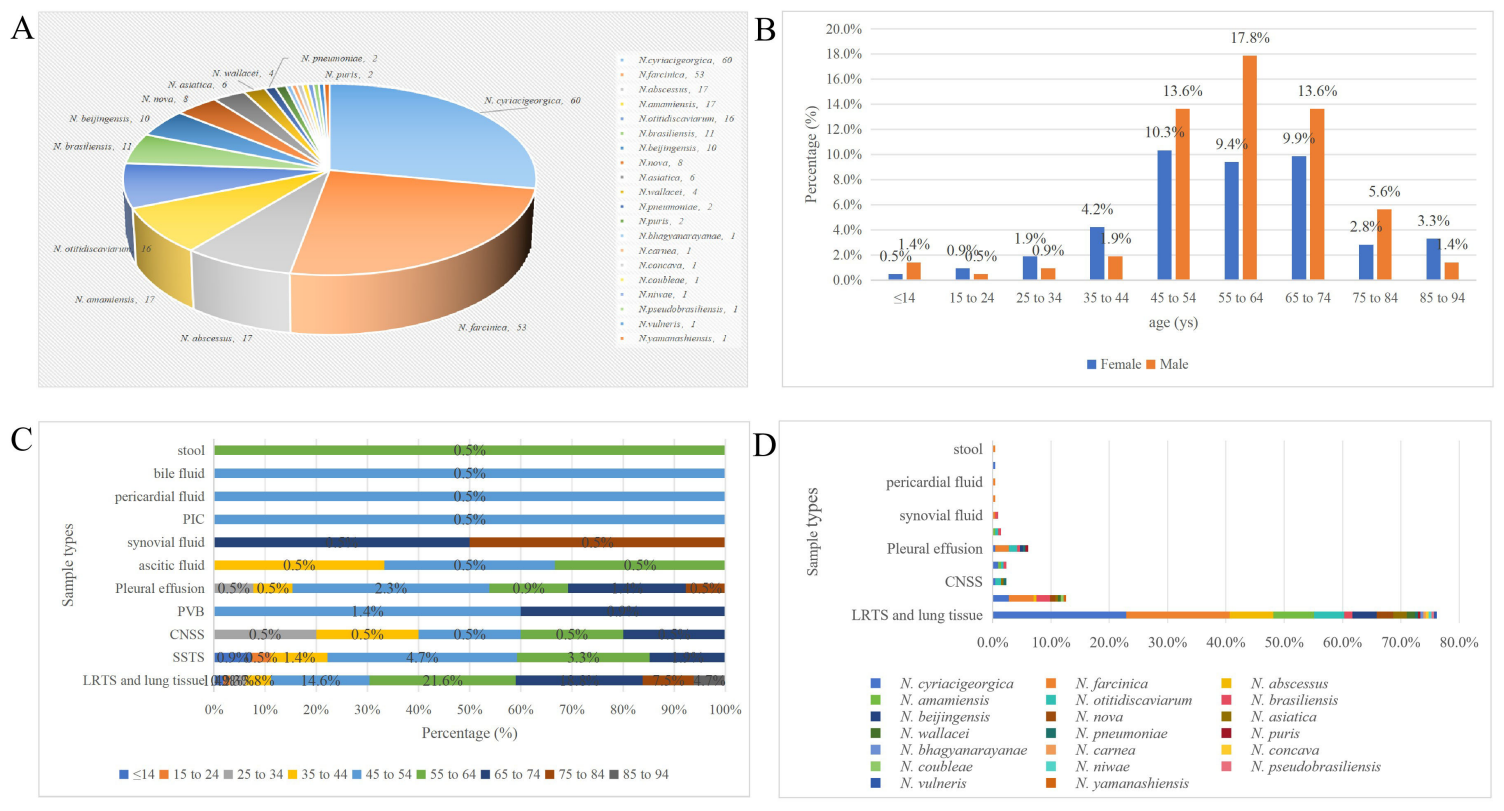
Demographic features of *Nocardia* isolates and nocardiosis patients. **(A)** Species distribution of 214 *Nocardia* isolates. **(B)** Correlation between ages and genders of the enrolled nocardiosis patients. **(C)** Correlation between ages and sample types of *Nocardia* spp. **(D)** Correlation between the commonly isolated *Nocardia* species and sample types. LRTS, Lower respiratory tract specimens: SSTS, skin and soft tissue specimens; CNSS, central nervous system specimens; PVB, peripheral venous blood; PIC, peripheral intravenous catheter.

Among the 214 *Nocardia* isolates, 20 different species were identified, including *N. cyriacigeorgica* (60, 28.0%), *N. farcinica* (53, 24.8%), *N. abscessus* (17, 7.9%), *N. amamiensis* (17, 7.9%), *N. otitidiscaviarum* (16, 7.5%), *N. brasiliensis* (11, 5.1%), *N. beijingensis* (10, 4.7%), *N. nova* (8, 3.7%), *N. asiatica* (6, 2.8%), and *N. wallacei* (4, 1.9%), respectively. These 10 *Nocardia* species constituted 94.4% (202 of 214) of all collected isolates. Furthermore, 12 strains for the rare *Nocardia* species were showed in **Figure 3**. Here, 3 isolates were misidentified by MALDI-TOF MS as *Nocardia*, but 16S rRNA sequencing showed 2 strains to be *Micromonospora chokoriensis* and 1 strain to be *Saccharopolyspora hordei*, Additionally, we also had provided a comparison analysis of MALDI-TOF MS and 16S rRNA sequencing results for these difficult-to-identify isolates in the **Supplementary Material** (**Supplementary Table S1**).

### Antibiotic susceptibility profiles

The susceptibility profil to 15 antibiotics for 214 *Nocardia* strains are summarized in **Table 3**, showing the MIC_50_, MIC_90_, the range, or S/I/R (%) for each antibiotic. All *Nocardia* strains were 100% susceptible to LZD, followed by AMK 99.5% (1 of 60 N*. cyriacigeorgica* was AMK-intermediate) and TMP-SMX 99.1% (2 resistant strains belonged to *N. farcinica*). For β-lactam antibiotics, including AMC, CRO, FEP, FOX and IPM, almost all data demonstrated a poor performance against *Nocardia* strains, whereas CRO demonstrated 100% susceptibility to *N. abscessus*, and high heterogeneity between *Nocardia* species, as shown in **Table 3**. For TOB, *N. amamiensis*, *N. brasiliensis*, and *N. beijingensis* were 100% sensitive, but the resistance rate for *N. farcinica* were 84.9%. However, for macrolides, 69.6% *Nocardia* strains were nonsusceptible to CLR. For quinolone antibiotics, CIP had a high resistance rate 62.2% against all *Nocardia* species, and the resistance rate to MFX was as high as 36.9%. For tetracyclines, DOX and MNO-resistant *Nocardia* accounted for 8.9% and 3.7%, respectively, but the intermediate rates were relatively high: 47.2% and 53.3%, respectively. TGC showed low MIC values against different *Nocardia* species, with its MIC_50_ and MIC_90_ at 0.5 and 2 mg/mL, respectively.

**Table 3:** Antimicrobial susceptibility and minimum inhibitory concentration (MIC) values (μg/mL) for 214 *Nocardia* isolates.

Drugs	Break -point		Species, no. of strains (%)													
			*N. cyriacigeorgica*	*N. farcinica*	*N. abscessus*	*N. amamiensis*	*N. otitidiscaviarum*	*N. brasiliensis*	*N. beijingensis*	N. Nova *	N. asiatica *	N. wallacei *	N. pneumoniae *	N. puris *	Other Nocardias *	All *Nocardias*
			60 (28.0)	53 (24.8)	17 (7.9)	17 (7.9)	16 (7.5)	11 (5.2)	10 (4.7)	8 (3.7)	6 (2.8)	4 (2.0)	2 (0.9)	2 (0.9)	8 (3.7)	214 (100)
TMP-SMX	S≤2/38, R≥4/76	MIC_50_	<=0.25	0.5	<=0.25	<=0.25	1	<=0.25	<=0.25	<=0.25	<=0.25	<=0.25			<=0.25	<=0.25
		MIC_90_	0.5	2	0.5	<=0.25	1	0.5	<=0.25	0.5	<=0.25	<=0.25			0.5	1
		Range	<=0.25 to 1	<=0.25 to >8	<=0.25 to 2	<=0.25 to 1	<=0.25 to 2	<=0.25 to 0.5	<=0.25 to <=0.25	<=0.25 to 1	<=0.25 to 0.5	<=0.25 to <=0.25			<=0.25 to 2	<=0.25 to >8
		S/I/R (%)	100/0/0	***96.2***/0/3.8	100/0/0	100/0/0	100/0/0	100/0/0	100/0/0	100/0/0	100/0/0	100/0/0	100/0/0	100/0/0	100/0/0	99.1/0/0.9
LZD	S≤8	MIC_50_	<=1.0	2	<=1.0	<=1.0	<=1.0	<=1.0	<=1.0	<=1.0	<=1.0	<=1.0			<=1.0	<=1.0
		MIC_90_	2	4	<=1.0	<=1.0	2	2	<=1.0	2	<=1.0	<=1.0			<=1.0	2
		Range	<=1.0 to 8	<=1.0 to 4	<=1.0 to 2	<=1.0 to <=1.0	<=1.0 to 2	<=1.0 to 2	<=1.0 to <=1.0	<=1.0 to 2	<=1.0 to <=1.0	<=1.0 to <=1.0			<=1.0 to 2	<=1.0 to 8
		S/NS (%)	100/0	100/0	100/0	100/0	100/0	100/0	100/0	100/0	100/0	100/0	100/0	100/0	100/0	100/0
AMC	S≤8/4, R≥32/16	MIC_50_	32	8	4	16	>64	8	>64	>64	>64	16			8	16
		MIC_90_	64	16	>64	32	>64	8	>64	>64	>64	16			64	>64
		Range	4 to >64	4 to 32	<=2.0 to >64	<=2.0 to 64	32 to >64	4 to 16	<=2.0 to >64	8 to >64	>64 to >64	8 to 16			<=2.0 to >64	<=2.0 to >64
		S/I/R (%)	10/35/55	***77.4***/17.0/5.6	**76.5**/0/23.5	41.2/41.2/17.6	0/0/100	***90.9***/9.1/0	20/10/70	12.5/0/87.5	0/0/100	25/75/0	50/0/50	0/50/50	50/12.5/37.5	40.2/20.6/39.2
CRO	S≤8, R≥64	MIC_50_	8	64	<=4.0	<=4.0	>64	8	<=4.0	8	<=4.0	>64			16	8
		MIC_90_	16	>64	<=4.0	<=4.0	>64	64	16	32	<=4.0	>64			64	>64
		Range	<=4.0 to >64	8 to >64	<=4.0 to <=4.0	<=4.0 to 16	32 to >64	<=4.0 to 64	<=4.0 to 16	<=4.0 to >64	<=4.0 to <=4.0	16 to >64			<=4.0 to >64	<=4.0 to >64
		S/I/R (%)	71.7/23.3/5.0	3.8/32.1/64.1	100/0/0	94.1/5.9/0	0/18.8/81.2	54.6/9.1/36.4	80/20/0	75/12.5/12.5	100/0/0	***0***/25/75	100/0/0	100/0/0	37.5/37.5/25	50.9/21/28.1
FEP		MIC_50_	8	>32	8	8	>32	32	8	8	4	>32			32	32
		MIC_90_	32	>32	16	16	>32	>32	16	32	8	>32			>32	>32
		Range	<=1.0 to >32	16 to >32	<=1.0 to >32	<=1.0 to >32	16 to >32	8 to >32	<=1.0 to 16	2 to 32	<=1.0 to 16	8 to >32			<=1.0 to >32	<=1.0 to >32
FOX		MIC_50_	128	64	<=4.0	8	>128	128	8	64	8	>128			64	64
		MIC_90_	>128	>128	8	32	>128	>128	16	128	8	>128			>128	>128
		Range	<=4.0 to >128	16 to >128	<=4.0 to 32	<=4.0 to 64	64 to >128	128 to >128	<=4.0 to 64	16 to 128	<=4.0 to 16	64 to >128			<=4.0 to >128	<=4.0 to >128
IMP	S≤4, R≥16	MIC_50_	4	4	32	4	64	>64	4	8	<=2.0	32			8	8
		MIC_90_	16	16	>64	>64	>64	>64	64	>64	4	32			>64	>64
		Range	<=2.0 to >64	<=2.0 to 64	<=2.0 to >64	<=2.0 >64	8 to >64	8 to >64	<=2.0 to >64	<=2.0 to >64	<=2.0 to 32	32 to 64			<=2.0 to >64	<=2.0 to >64
		S/I/R (%)	56.7/18.3/25	54.7/24.5/20.8	17.7/11.8/70.5	70.6/0/29.4	0/6.3/93.7	0/18.2/81.8	50/10/40	***37.5***/12.5/50	83.3/0/16.7	0/0/100	50/0/50	100/0/0	37.5/12.5/50	45.3/15/39.7
AMK	S≤8, R≥16	MIC_50_	<=1.0	<=1.0	<=1.0	<=1.0	<=1.0	<=1.0	<=1.0	<=1.0	<=1.0	<=1.0			<=1.0	<=1.0
		MIC_90_	<=1.0	<=1.0	<=1.0	<=1.0	<=1.0	<=1.0	<=1.0	<=1.0	<=1.0	2			<=1.0	<=1.0
		Range	<=1.0 to 32	<=1.0 to 4	<=1.0 to <=1.0	<=1.0 to 2	<=1.0 to <=1.0	<=1.0 to 2	<=1.0 to <=1.0	<=1.0 to <=1.0	<=1.0 to <=1.0	<=1.0 to 8			<=1.0 to 4	<=1.0 to 32
		S/I/R (%)	***98.3***/1.7/0	100/0/0	100/0/0	100/0/0	100/0/0	100/0/0	100/0/0	100/0/0	100/0/0	100/0/0	100/0/0	100/0/0	100/0/0	99.5/0.5/0
TOB	S≤4, R≥16	MIC_50_	<=1.0	16	<=1.0	<=1.0	4	<=1.0	<=1.0	>16	<=1.0	>16			<=1.0	<=1.0
		MIC_90_	<=1.0	>16	2	<=1.0	16	<=1.0	<=1.0	>16	<=1.0	>16			2	>16
		Range	<=1.0 to >16	8 to >16	<=1.0 to 8	<=1.0 to <=1.0	<=1.0 to >16	<=1.0 to <=1.0	<=1.0 to <=1.0	<=1.0 to >16	<=1.0 to 2	<=1.0 to >16			<=1.0 to 8	<=1.0 to >16
		S/I/R (%)	***95***/0/5	0/15.1/84.9	88.2/11.8/0	100/0/0	***56.3***/25/18.7	100/0/0	100/0/0	12.5/0/87.5	100/0/0	25/0/75	100/0/0	100/0/0	87.5/12.5/0	64.5/7.0/28.5
CLR	S≤2, R≥8	MIC_50_	8	>16	8	0.12	>16	>16	1	<=0.06	8	4			2	8
		MIC_90_	>16	>16	>16	4	>16	>16	2	0.12	>16	8			16	>16
		Range	0.25 to >16	4 to >16	<=0.06 to >16	<=0.06 to >16	16 to >16	2 to >16	0.25 to 4	<=0.06 to 0.12	1 to >16	4 to 8			<=0.06 to >16	<=0.06 to >16
		S/I/R (%)	33.4/13.3/53.3	0/1.9/98.1	29.4/5.9/64.7	76.5/11.8/11.7	0/0/100	18.2/0/81.8	90/10/0	100/0/0	33.3/0/66.7	0/50/50	100/0/0	0/0/100	50/12.5/37.5	30.4/7.5/62.1
CIP	S≤1, R≥4	MIC_50_	>4	1	4	>4	4	4	>4	>4	>4	1			2	4
		MIC_90_	>4	4	>4	>4	>4	>4	>4	>4	>4	1			4	>4
		Range	0.5 to >4	0.25 to 4	<=0.12 to >4	4 to >4	2 to >4	2 to >4	0.5 to >4	>4 to >4	>4 to >4	0.5 to >4			0.25 to >4	<=0.12 to >4
		S/I/R (%)	5/15/80	56.6/30.2/13.2	17.7/0/82.3	0/0/100	0/31.3/68.7	0/36.4/63.6	20/0/80	0/0/100	0/0/100	***75***/0/25	0/0/100	0/0/100	37.5/37.5/25	20.6/17.3/62.1
MFX	S≤1, R≥4	MIC_50_	2	0.5	4	8	2	1	>8	4	>8	<=0.25			0.5	2
		MIC_90_	4	1	8	>8	4	1	>8	4	>8	<=0.25			1	8
		Range	1 to >8	<=0.25 to 2	<=0.25 to >8	2 to >8	0.5 to 4	0.5 to 2	<=0.25 to >8	2 to 4	>8 to >8	<=0.25 to 8			<=0.25 to 4	<=0.25 to >8
		S/I/R (%)	23.4/38.3/38.3	88.7/11.3/0	17.7/17.6/64.7	0/5.9/94.1	25/50/25	100/0/0	20/0/80	0/0/100	0/0/100	75/0/25	0/0/100	0/100/0	87.5/0/12.5	42.1/21/36.9
MNO	S≤1, R≥8	MIC_50_	2	2	<=1.0	<=1.0	2	2	<=1.0	4	<=1.0	<=1.0			<=1.0	2
		MIC_90_	4	4	<=1.0	<=1.0	2	2	<=1.0	8	<=1.0	<=1.0			4	4
		Range	<=1.0 to >8	<=1.0 to 4	<=1.0 to 4	<=1.0 to 2	<=1.0 to 4	<=1.0 to 4	<=1.0 to <=1.0	<=1.0 to >8	<=1.0 to <=1.0	<=1.0 to <=1.0			<=1.0 to 8	<=1.0 to >8
		S/I/R (%)	30/63.3/6.7	7.6/92.4/0	88.2/11.8/0	94.1/5.9/0	37.5/62.5/0	36.4/63.6/0	100/0/0	12.5/50/37.5	100/0/0	100/0/0	50/50/0	100/0/0	62.5/25/12.5	43/53.3/3.7
DOX	S≤1, R≥8	MIC_50_	2	4	0.25	<=0.12	1	4	<=0.12	8	<=0.12	0.25			0.25	2
		MIC_90_	4	8	0.5	0.5	2	4	1	16	0.5	0.25			4	4
		Range	0.25 to 8	1 to 8	<=0.12 to 2	<=0.12 to 0.5	0.25 to 4	1 to 8	<=0.12 to 1	8 to 16	<=0.12 to 1	<=0.12 to 0.25			<=0.12 to 8	<=0.12 to 16
		S/I/R (%)	31.7/66.7/1.6	5.7/79.3/15.0	94.1/5.9/0	100/0/0	50/50/0	27.3/63.6/9.1	100/0/0	0/0/100	100/0/0	100/0/0	100/0/0	100/0/0	50/37.5/12.5	43.9/47.2/8.9
TGC		MIC_50_	0.25	1	0.25	0.12	0.25	0.25	0.5	2	0.25	0.25			0.5	0.5
		MIC_90_	1	2	0.5	1	0.5	0.5	1	4	0.5	0.25			1	2
		Range	0.06 to 1	0.06 to >4	0.06 to 1	0.03 to 1	0.12 to 0.5	0.06 to 0.5	0.06 to 1	0.25 to 4	0.12 to 0.5	0.12 to 0.5			0.12 to 2	0.03 to >4
MDR, XDR, PDR			38.3, 0, 0	24.5, 0, 0	23.5, 0, 0	17.7, 0, 0	68.8, 0, 0	18.2, 0, 0	0, 0, 0	1/8, 0, 0	4/6, 0, 0	3/4, 0, 0	1/2, 0, 0	1/2, 0, 0	3/8, 0, 0	32.2, 0, 0

TMP-SMX, Trimethoprim-Sulfamethoxazole; LZD, Linezolid; AMK, Amikacin; TOB, Tobramycin; AMC, Amoxicillin-clavulanic acid; FOX, Cefoxitin; CRO, Ceftriaxone; FEP, Cefepime; IPM, Imipenem; CLR, Clarithromycin; CIP, Ciprofloxacin; MFX, Moxifloxacin; MNO, Minocycline; DOX, Doxycycline; TGC, Tigecycline; S, susceptible; I, intermediate; R, resistant; NS, non-susceptible; MIC_50_ and MIC_90_, MICs at which 50% and 90% of the strains were inhibited, respectively.

MDR, non-susceptibility >1 agent in >= 3 antimicrobial categories. XDR, non-susceptibility >1 agent in all but <= 2 categories. PDR, non-susceptibility to all antibiotics. The defining is excluding the antimicrobial affected by no-acquired resistance for each drug pattern type.

Red italic bold font, the susceptibility (%) of 7 *Nocardia* species in our study that showed differences from the Expected Antimicrobial Susceptibility Patterns (CLSI M24S-Ed2).

^*^Owing to the small number of isolates for these Nocardia species, the corresponding S/I/R (%) and MDR (%) values are not representative.

The antibiotic resistance profiles varied within different *Nocardia* species. The susceptibility rate of *N. cyriacigeorgica* to AMK (98.3%), TOB (95%), and CRO (71.7%) had decreased. The *N. farcinica* had a 0% sensitivity rate to CLR and TOB, and showed extensive resistance to β-lactam antibiotics (CRO, 3.8%), quinolones (CIP, 55.6%), and tetracyclines (MNO, 7.6%). The *N. amamiensis*, as an independent species in *Nocardia* genus, was fully resistant to quinolones and had a high resistance rate to β-lactam antibiotics. It was worth noting that the resistance phenotypes of *N. abscessus* and *N. beijingensis* were analyzed in our study separately, which were studied usually as the *N. abscessus complex* in other studies (Hamdi et al., 2020; Wang et al., 2022; Yang J. et al., 2023; Han et al., 2024). They are both founded 100% sensitive to TMP-SMX, LZD, and AMK, however, there were significant differences in resistance rates to other antibiotics excluding quinolones. Moreover, except for *N. beijingensis*, other strains always had poor activity to CLR. Additionally, 8 different rare *Nocardia* strains with only 1 isolate per specie were combined into the category of “Other *Nocardia*” to analyse their overall sensitivity rate, as shown in **Table 3**.

The antimicrobial susceptibility patterns in our study were compared with those provided by CLSI standard M24S-Ed2 (Parrish et al., 2023). In **Table 3**, a strong correlation between the drug pattern types and *Nocardia* species identified was illustrated. Nevertheless, some variations were highlighted in red italic bold font of **Table 3**. Especially the following 7 species of *Nocardia:* for *N. cyriacigeorgica* isolates, the sensitivity rate of CRO, TOB and AMK were 71.7%, 95% and 98.3%, respectively, despite the drug pattern indicating sensitivity. And the sensitivity rate of AMC and TMP-SMX in *N. farcinica* isolates were 77.4% and 96.2%, whose drug patterns were determined to be susceptible. The sensitivity rate of AMC in *N. abscessus* isolates was only 76.5%, and similarly, the sensitivity rate of AMC in *N. brasiliensis* was 90.9%. The *N. otitidiscaviarum* isolates, the sensitivity rate of TOB was 56.3%.

**Figure 4:**
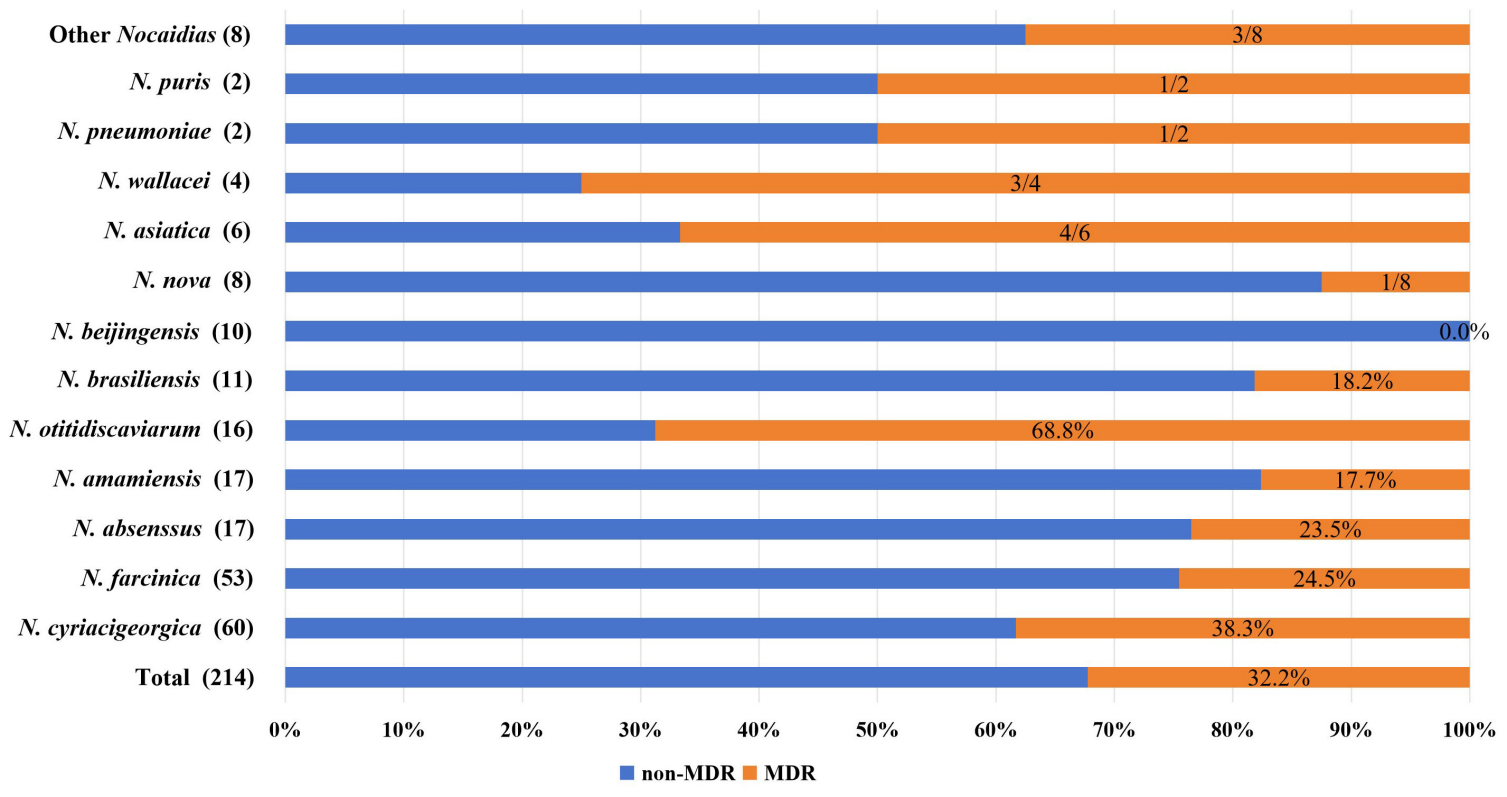
The proportion of MDR strains of 214 *Nocardia* isolates.

As described by Valdezate S (Valdezate et al., 2017) in the definition of MDR, the detection rates of MDR, XDR, and PDR for *Nocardia* species with assigned drug patterns varied due to the acquired resistance of the strains. To avoid statistical bias, we showed “n (MDR isolates)/n (total isolates)” as MDR ratio for *Nocardia* species (n < 10). **Table 3** and **Figure 4** showed us in descending order as follows: 68.8% for *N. otitidiscaviarum*, 38.3% for *N. cyriacigeorgica*, 24.5% for *N. farcinic*a, 23.5% for *N. abscessus*, 18.2% for *N. brasiliensis*, 3/4 for *N. wallacei* and 1/8 for *N. nova*. For species without a corresponding susceptibility pattern, MDR values ranged from 17.7% for *N. amamiensis* to 4/6 for *N. asiatica*. No strains exhibited the extensively XDR and PDR phenotypes.

### Detection of antibiotic resistance genes and virulence genes

Given the significant differences in resistance phenotypes between the 7 species of *Nocardia* (51 strains) mentioned above and the previously published resistance patterns in CLSI standard M24S-Ed2 (Parrish et al., 2023), WGS was used to analyze whether these 51 strains carried antibiotic resistance and virulence genes. The sequences were annotated for gene functions by aligning against the Comprehensive Antibiotic Resistance Database (CARD) antibiotic resistance gene database and the pathogen virulence factor database (VFDB) using the Diamond program (Yang et al., 2025). The gene results were typed by aligning against the PUBMLST database (https://pubmlst.org) using the MLST (version 2.19.0) program.

**Figure 5:**
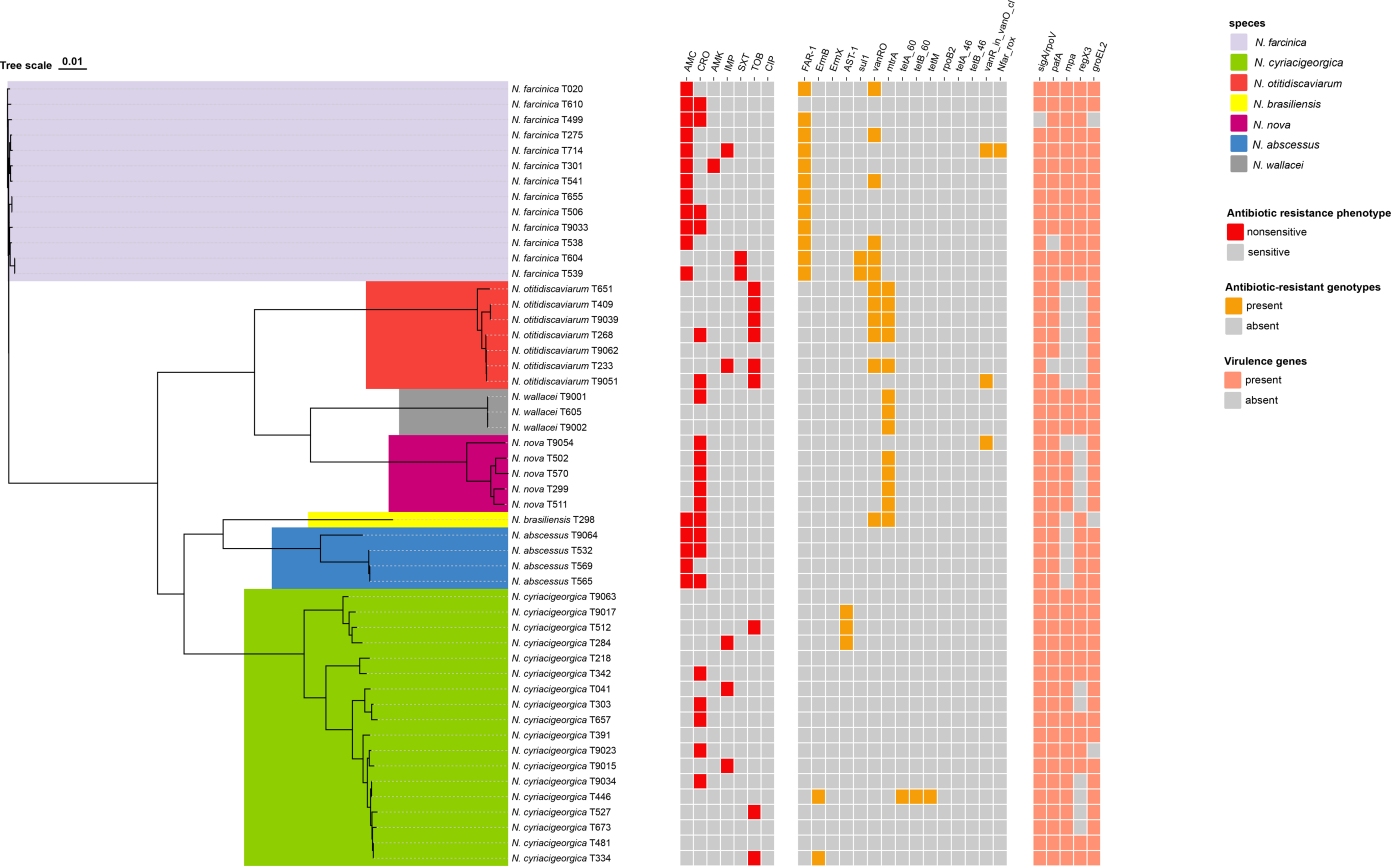
Distribution of antibiotic resistance genes and virulence genes of 7 *Nocardia* species (51 strains). The main associations between drug-resistant phenotypes, resistance genes, and virulence genes are marked in red, orange, and pink, respectively.

As shown in **Figure 5**, 12 antibiotic resistance genes (such as *AST-1*, *FAR-1*, *sul1*, *mtrA*) were identified. The *AST-1* gene was present in *N. cyriacigeorgica* isolates associated with resistance to AMC, non-sensitivity to CRO, and could also explain the elevated MIC of FEP; the *FAR-1* gene was 92.3% present in *N. farcinica* isolates, which was associated with resistance to AMC, CRO, IPM and the elevated MIC of FEP. The *sul1* gene was present in *N. farcinica* isolates to explain the resistance to TMP-SMX. The *mtrA* gene was found in *N. otitidiscaviarum*, *N. brasiliensis*, *N. nova* and *N. wallacei*, and could encode a transcriptional activator of a multidrug efflux pump, and also confer resistance to a variety of antibiotics, including β-lactams, rifampicin, and macrolides. These genes encode a variety of resistance mechanisms, including efflux pumps, β-lactamases, and plasmid-mediated methyltransferases, which confer resistance through antibiotic inactivation, active efflux, and target modification (**Supplementary Table S2**). No potential resistance genes were detected in the *N. abscessus*.

A total of 11 virulence genes were detected, including *sigA/rpoV*, *ahpC*, *plr/gapA*, *pafA*, *mpa*, *regX3*, *groEL2*, etc. These virulence genes were involved in promoting bacterial adhesion, invasion, and survival within macrophages, or in regulating the expression of other virulence factors, or in enhancing bacterial survival and adaptation to environmental stress within host cells through protein degradation mechanisms, thereby enhancing the pathogenicity of the 7 *Nocardia* species. More details were provided in **Supplementary Table S3**.

### Treatment and outcomes

In the 213 nocardiosis cases, treatment details were available for 202 cases, while 10 cases were missing of the treatment plans, and 1 case was considered to be *Nocardia* colonization without targeted treatment. Of 202 cases, 53.5% (108 of 202) patients received TMP-SMX monotherapy or with fluoroquinolones or β-lactam antibiotics or LZD multidrug regimen, 85.6% (173 of 202) patients had clinical improvement and 14.4% (29 of 202) failed.

Based on the data presented in **Table 4**, there were 164 cases with simple pulmonary infection, 14.6% (24 of 164) patients received TMP-SMX monotherapy, 15.2% (25 of 164) received TMP-SMX plus β-lactam antibiotics combination regimen, 11.0% (18 of 164) received TMP-SMX plus LZD combination regimen, 4.9% (8 of 164) received TMP-SMX plus fluoroquinolones antibiotics combination regimen, 11.0% (18 of 164) received triple therapy regimen, and 43.3% (71 of 164) received other antibiotics regimens. Furthermore, 84.8% (139 of 164) patients had clinical improvement and 15.2% (25 of 164) failed.

**Table 4:** Information on treatment and outcomes of 202 nocardiosis patients presenting with different types of infections and species.

Infection Types (strains)	Species (n)	Treatment	Number	Outcomes
Simple pulmonary infection (164)	*N. cyriacigeorgica* (48)	TMP-SMX monotherapy	9	Recovered (8), Failure (1)
		TMP-SMX with β-lactam antibiotics	7	Recovered (6), Failure (1)
		TMP-SMX with linezolid	9	Recovered (8), Failure (1)
		TMP-SMX with Quinolone antibiotics	1	Recovered (1)
		Triple Therapy	6	Recovered (5), Failure (1)
		Moxifloxacin monotherapy	5	Recovered (4), Failure (1)
		IMP/MEM monotherapy	4	Recovered (3), Failure (1)
		Other antibiotics	7	Recovered (7)
	*N. farcinica* (39)	TMP-SMX monotherapy	6	Recovered (6)
		TMP-SMX with β-lactam antibiotics	6	Recovered (5), Failure (1)
		TMP-SMX with linezolid	1	Recovered (1)
		TMP-SMX with Quinolone antibiotics	4	Recovered (3), Failure (1)
		Triple Therapy	3	Recovered (2), Failure (1)
		Moxifloxacin monotherapy	6	Recovered (6)
		β-lactam antibiotic monotherapy	4	Recovered (3), Failure (1)
		Other antibiotics	9	Recovered (7), Failure (2)
	*N. amamiensis* (16)	TMP-SMX monotherapy	2	Recovered (1), Failure (1)
		β-lactam antibiotic monotherapy	2	Recovered (2)
		Quinolone antibiotic monotherapy	1	Recovered (1)
		linezolid monotherapy	1	Recovered (1)
		TMP-SMX with linezolid	3	Recovered (1), Failure (2)
		TMP-SMX with β-lactam antibiotics	3	Recovered (3)
		Triple Therapy	2	Failure (2)
		Other antibiotics	2	Recovered (2)
	*N. abscessus* (14)	TMP-SMX monotherapy	4	Recovered (4)
		TMP-SMX with β-lactam antibiotics	1	Recovered (1)
		TMP-SMX with linezolid	1	Failure (1)
		TMP-SMX with Quinolone antibiotics	2	Recovered (1), Failure (1)
		β-lactam antibiotic monotherapy	3	Recovered (3)
		Other antibiotics	3	Recovered (3)
	*N. otitidiscaviarum* (13)	TMP-SMX monotherapy	1	Recovered (1)
		β-lactam antibiotic monotherapy	1	Recovered (1)
		Quinolone antibiotic monotherapy	2	Recovered (2)
		linezolid monotherapy	1	Recovered (1)
		TMP-SMX with linezolid	1	Recovered (1)
		TMP-SMX with Quinolone antibiotics	1	Recovered (1)
		TMP-SMX with β-lactam antibiotics	1	Failure (1)
		Triple Therapy	2	Recovered (2)
		Other antibiotics	3	Recovered (3)
	*N. beijingensis* (9)	TMP-SMX monotherapy	1	Recovered (1)
		linezolid monotherapy	2	Recovered (2)
		β-lactam antibiotic monotherapy	2	Recovered (1), Failure (1)
		TMP-SMX with Quinolone antibiotics	1	Recovered (1)
		TMP-SMX with β-lactam antibiotics	3	Recovered (2), Failure (1)
	*N. nova* (5)	TMP-SMX monotherapy	1	Recovered (1)
		IPM with AMK	1	Failure (1)
		Triple Therapy	1	Recovered (1)
		Other antibiotics	2	Recovered (2)
	*N. asiatica* (5)	TMP-SMX with β-lactam antibiotics	2	Recovered (2)
		TMP-SMX with linezolid	1	Recovered (1)
		Triple Therapy	2	Recovered (1), Failure (1)
	*N. brasiliensis* (3)	linezolid monotherapy	1	Recovered (1)
		TMP-SMX with linezolid	1	Recovered (1)
		IPM with TGC	1	Recovered (1)
	*N. wallacei* (2)	Aminoglycoside with β-lactam antibiotics	1	Recovered (1)
		Triple Therapy	1	Recovered (1)
	*N. puris* (2)	Quinolone antibiotic monotherapy	1	Recovered (1)
		Triple Therapy	1	Recovered (1)
	*N. bhagyanarayanae* (1)	β-lactam antibiotic monotherapy	1	Recovered (1)
	*N. carnea* (1)	IPM with TGC	1	Recovered (1)
	*N. concava* (1)	MOX with linezolid	1	Recovered (1)
	*N. niwae* (1)	TMP-SMX with β-lactam antibiotics	1	Recovered (1)
	*N. pneumoniae* (1)	Aminoglycoside with β-lactam antibiotics	1	Recovered (1)
	*N. pseudobrasiliensis* (1)	AMK with MEM	1	Recovered (1)
	*N. vulneris* (1)	TMP-SMX with β-lactam antibiotics	1	Failure (1)
	*N. yamanashiensis* (1)	β-lactam antibiotic monotherapy	1	Recovered (1)
Simple skin infection (20)	*N. farcinica* (6)	TMP-SMX monotherapy	1	Recovered (1)
		TMP-SMX with Quinolone antibiotics	1	Recovered (1)
		Aminoglycoside with β-lactam antibiotics	1	Recovered (1)
		Other antibiotics	1	Recovered (1)
		Debridement	1	Recovered (1)
	*N. brasiliensis* (5)	Quinolone antibiotic monotherapy	1	Recovered (1)
		TMP-SMX with linezolid	1	Recovered (1)
		Other antibiotics	2	Recovered (2)
		Debridement	1	Recovered (1)
	*N. cyriacigeorgica* (3)	β-lactam antibiotic monotherapy	1	Recovered (1)
		linezolid with LEV	1	Recovered (1)
		Triple Therapy	1	Recovered (1)
	*N. nova* (2)	β-lactam antibiotic monotherapy	1	Recovered (1)
		Triple Therapy	1	Recovered (1)
	*N. abscessus* (2)	TMP-SMX with β-lactam antibiotics	1	Recovered (1)
		Other antibiotics	1	Recovered (1)
	*N. coubleae* (1)	Triple Therapy	1	Recovered (1)
	*N. wallacei* (1)	Triple Therapy	1	Recovered (1)
Simple intra-abdominal infection (2)	*N. cyriacigeorgica* (1)	Quinolone antibiotic monotherapy	1	Recovered (1)
	*N. amamiensis* (1)	β-lactam antibiotic monotherapy	1	Recovered (1)
Simple bloodstream infection (1)	*N. farcinica* (1)	LEV with MEM	1	Recovered (1)
Pulmonary infection and skin infection (6)	*N. farcinica* (3)	TMP-SMX with Quinolone antibiotics	1	Recovered (1)
		β-lactam antibiotic monotherapy	1	Recovered (1)
		Triple Therapy	1	Recovered (1)
	*N. brasiliensis* (2)	β-lactam antibiotic monotherapy	1	Recovered (1)
		TMP-SMX with β-lactam antibiotics	1	Recovered (1)
	*N. cyriacigeorgica* (1)	TMP-SMX with linezolid	1	Recovered (1)
Pulmonary infection and intracranial infection (5)	*N. otitidiscaviarum* (2)	β-lactam antibiotic monotherapy	1	Failure (1)
		Triple Therapy	1	Failure (1)
	*N. cyriacigeorgica* (1)	β-lactam antibiotic monotherapy	1	Recovered (1)
	*N. asiatica* (1)	MOX with MEM	1	Recovered (1)
	*N. pneumoniae* (1)	Quinolone antibiotic monotherapy	1	Failure (1)
Pulmonary infection and cardiovascular infection (1)	*N. farcinica* (1)	β-lactam antibiotic monotherapy	1	Recovered (1)
Pleural infection and intra-abdominal infection (1)	*N. otitidiscaviarum* (1)	β-lactam antibiotic monotherapy	1	Failure (1)
multiple-site infection (2)	*N. cyriacigeorgica* (1)	TMP-SMX with linezolid	1	Recovered (1)
	*N. farcinica* (1)	TMP-SMX with linezolid	1	Recovered (1)

Of the 20 patients with simple skin and soft tissue infections, 20% (4 0f 20) patients received TMP-SMX monotherapy or with fluoroquinolones or β-lactam antibiotics or LZD combination regimen, 25% (5 of 20) patients received LZD, fluoroquinolones, and β-lactam antibiotics monotherapy or combination regimen, 20% (4 of 20) received triple therapy regimen, and 10% (2 of 20) received debridement. Furthermore, no case was failed.

## Discussion

This is the largest study to date in Henan Province, China, that correlates antimicrobial susceptibility with molecular identification and resistance mechanisms of *Nocardia* species. As expected, in our study, the most common source of positive *Nocardia* cultures was the lower respiratory tract (76.1% of isolates), following by the skin and soft tissue system (12.7% of isolates), which is basically consistent with the clinical distribution in other large domestic and international *Nocardia*-related research case series (Mootsikapun et al., 2005; Huang et al., 2019a; Huang et al., 2019b; Lebeaux et al., 2019; Tan et al., 2020; Yeoh et al., 2022; Hershko et al., 2023; Yang J. et al., 2023; Han et al., 2024; Di et al., 2025). However, there are also studies with inconsistent detection rates, such as a study from Taiwan (Lao et al., 2022), which found that cutaneous nocardiosis was the most common (50%) and pulmonary nocardiosis was the second most common clinical form (35.3%). These may be due to differences in the sources of *Nocardia* - positive specimens caused by geographical and climatic differences, affected population or sampling bias in the studies.

*Nocardia* species grow slowly and exhibit weak biochemical reactivity. In clinical microbiology laboratories, the identification of these species primarily relies on MALDI-TOF MS. However, molecular sequencing techniques, such as 16S rRNA sequencing, are still required for the identification of certain species (Rodríguez-Temporal et al., 2023; Sharma et al., 2024). In our study, 75.7% (162 of 214) *Nocardia* isolates were accurately identified to the species level by MALDI-TOF MS. In a study of Body BA, 76% of *Nocardia* strains were correctly identified to the species using the MALDI-TOF MS, which is comparable to our findings (Body et al., 2018). As shown in **Supplementary Table S1**, among the 55 strains verified by 16S rRNA, 94.5% (52 of 55) were identified as *Nocardia* by MALDI-TOF MS with not being correctly differentiated to the species level, but 5.5% (3 of 55) reported as *Nocardia* were ultimately identified as non-*Nocardia* species by 16S rRNA sequencing, which was lower than Ölmez S’ findings with the misidentification rate 36.8% (7 of 19) for MS (Ölmez et al., 2023). Exploring the reasons: the agarophilic characteristics or the growth status of some strains such as *N. abscessus* and *N. amamiensis*, might lead to agar interference during protein extraction and then result in the incorrect identification by MALDI-TOF MS; *N. africana* and *N. nova*, which belong to the *Nocardia nova* complex with high homology in the 16S rRNA gene sequence, cannot be effectively distinguished by MALDI-TOF MS; for some rare *Nocardia* species, such as *N. carnea*, *N. yamanashiensis*, *N. bhagyanarayanae*, *N. niwae* and *N. coubleae*, the MALDI-TOF MS identification system lacked a more comprehensive identification database, therefore the successful identification was lower.

The 214 clinical *Nocardia* isolates included 20 different species. In the present study, *N. cyriacigeorgica*, *N. farcinica*, *N. abscessus* and *N. amamiensis* were the most frequently isolated ranking in the top three, which is somewhat different from the conclusions reported in other related studies (Huang et al., 2019b; Hamdi et al., 2020; Yeoh et al., 2022; Wang et al., 2023; Yang J. et al., 2023; Han et al., 2024). Moreover, *N. cyriacigeorgica* (30.2%) ranked first in pulmonary nocardiosis, while *N. farcinica* (33.3%) had the highest isolation rate in cutaneous nocardiosis. *Nocardia* is sporadically distributed worldwide, and the predominant pathogenic strains reported vary not only among different countries and regions but also among different areas within the same country. This variation may be associated with differences in geographical distribution, population distribution, climate, hospital levels, laboratory culture methods and capabilities, and the identification techniques employed.

Our data showed that the majority of patients were older, with an average age of 59 years, and there was a slight predominance of males, which was consistent with previous literature. In terms of occupational classification, farmers made up the majority (116, 54.5%), and the isolation rate of *Nocardia* in the Nanyang (60, 28.0%) area was the highest by a wide margin, followed by Zhengzhou (26, 12.15%), with the reasons of these patients, who would be more exposed to contaminated soil, and were more susceptible to environmental *Nocardia* infections. Furthermore, Nanyang Central Hospital as one of the participating centers, its specimen submission volume was second only followed that from the First Affiliated Hospital of Zhengzhou University. Consequently, the data primarily reflected the distribution of sample sources, thus, a wider range surveillance from other regions of Henan Province would be needed to obtain the true regional distribution. We also noted a steady increase in the number of specimens over the study period, which might indicate an increasing incidence, or more likely, enhanced awareness among clinicians of the need for *Nocardia* culture, identification, and AST, improved recognition of its pathogenicity, and easier access to these tests.

In this study, approximately 54% of *Nocardia* infection patients were immunocompetent. Studies (Fujita et al., 2016; Yang J. et al., 2023; Han et al., 2024) stated that immunocompetent patients with COPD or bronchiectasis especially if they were using oral or inhaled corticosteroids, would show a markedly increased risk of developing pulmonary nocardiosis. In chronic pulmonary diseases, corticosteroid therapy is believed to cause lower respiratory tract epithelial damage, which facilitates the colonization of *Nocardia* (Galar et al., 2021). Diabetes, chronic kidney disease, and cirrhosis are usually considered risk factors for invasive nocardiosis (Peleg et al., 2007). Our study showed that the most common underlying diseases in patients with *Nocardia* infection were acute or chronic lung disease (153, 71.8%), among these patients, the most common comorbidities, in sequence were pulmonary infection (119, 55.9%), bronchiectasis (33, 15.5%), and COPD (19, 8.9%). Yungang Han (Han et al., 2024) and Jing Yang (Yang J. et al., 2023) all reported that bronchiectasis was the most common comorbidity in patients with pulmonary nocardiosis, occurring in 54.9% and 15.4%, respectively. Yang CH’ study (Yang CH. et al., 2023) indicated that the most common comorbidities were diabetes and COPD, occurring in 30% and 26.7%, respectively. Ott (Ott et al., 2019) expounded that 58.1% of the patients had underlying pulmonary diseases, with COPD being the most common. These results differed from our study.

Patients with pulmonary nocardiosis usually have non - specific clinical manifestations, and most present with symptoms of pulmonary purulent infection. In present study, patients had cough (153, 71.8%), sputum production (148, 69.5%), with nearly half of them producing yellow - purulent sputum. Fever was the next most common symptom (128, 60.1%), all with moderate to high fever, fluctuating between 38.3 - 39.7°C. Chest pain, chest tightness, hemoptysis and fatigue were also common symptoms. On physical examination, the most frequent signs were dry/wet rales (76, 35.9%) and wheezing (14, 6.6%). Moreover, lesions often involved bilateral lungs (178, 83.6%) and were prone to involve the pleura (119, 55.9%). The most common radiological changes were patchy consolidation (116, 54.5%), nodular shadows (104, 48.8%), and various forms of high - density shadows. Because of the variable radiological appearance, it was often difficult to distinguish from tuberculosis, tumors, and other fungal infections, easily prone to misdiagnosis. Approximately one-third of patients will experience dissemination to other organs, with a preference for CNS as well as skin and soft tissues (Xue et al., 2024). Patients with skin involvement usually presented with subcutaneous abscesses (23, 10.8%), and systemic symptoms may not be obvious (Yeoh et al., 2022). Patients with compromised immune function were more susceptible to disseminated infection. Those founding CNS dissemination mainly presented with headache, meningeal irritation signs, and focal neurological deficits, with typical cranial MRI showing ring-enhancement of the lesions (Schlaberg et al., 2014). Since around 40% of cases with cerebral involvement may show asymptomatic, systematic cerebral MRI is mandatory in all nocardiosis cases (Van den Bogaart and Manuel, 2022). In laboratory tests, most patients had varying degrees of increased WBC, neutrophils, and inflammatory markers. Lymphocyte subset analysis can reflect the systemic inflammatory state of the body, also indicating that compromised immune function is a risk factor for nocardiosis (Yetmar et al., 2023a).

For the long-term treatment of *Nocardia* infections, it is often necessary to switch between various antibiotics or even use combination therapy. TMP-SMX and β-lactam antibiotics such as CRO and IPM are commonly used drugs for nocardiosis. LZD will be required if severe infections. Our study shows that LZD is 100% sensitive to *Nocardia* species, with the same sensitivity in most previous studies (Valdezate et al., 2017; Hamdi et al., 2020; Toyokawa et al., 2021; Wang et al., 2022; Yang J. et al., 2023; Han et al., 2024). Except for *N. farcinica* (96.2% sensitive to TMP-SMX), all other *Nocardia* species were 100% sensitive to TMP-SMX. And the sensitivity rates of *N. farcinica* to CRO and IPM are 3.8% and 54.7%, respectively, those might be related to the fact that the TMP-SMX resistance gene *sul1* and class A β-lactamase gene *FAR-1* were only identified in *N. farcinica* (Valdezate et al., 2015; Che et al., 2022; Nathar et al., 2024; Di et al., 2025). It was noteworthy that different *Nocardia* isolates carry different resistance genes, and the types and expression levels of resistance genes carried by the same *Nocardia* species may also vary. This might also explain why other research findings, such as Hamdi’s study, suggested that the sensitivity rate of *N. farcinica* to CRO is 3% and to IPM is 83% (Hamdi et al., 2020). Church D reported IPM resistance occurred only in strains of the *N. farcinica* and *N. nova* complex, while CRO resistance occurred only in the former (Church et al., 2025). Such similar explanations also apply to *N. cyriacigeorgica*, the sensitivity rate of CRO and IPM was 71.7% and 56.7%, respectively, the class A β-lactamase gene *AST-1* was only identified in *N. cyriacigeorgica*, consistent with previous study (Di et al., 2025). *N. abscessus* was 100% susceptible to CRO, but its sensitivity rate to IPM was only 17.7%, different from other studies (Schlaberg et al., 2014; Hamdi et al., 2020; Wang et al., 2022; Yang J. et al., 2023; Han et al., 2024), which showed a sensitivity rate of about 95% to CRO and a sensitivity rate of 31% - 64% to IPM. This might be one possible explanation that in our study, *N. abscessus*, *N. beijingensis*, and *N. asiatica* in *N. abscessus* complex were analyzed separately. Although no related resistance genes were detected in *N. abscessus*, all isolates carried virulent factors such as *ahpC*, *pafA*, *sigA/rpoV*, *regX3* and *groEL2* genes, related to stress survival, regulation, and adherence. In our study, *N. otitidiscaviarum* was 100% resistant to β-lactam antibiotics, CLR, and CIP, while Han Y reported that 100% resistant to β-lactam antibiotics and 20% susceptible to CLR and CIP (Han et al., 2024). Also in other studies, there were around 10% susceptible to β-lactam antibiotics, CLR and CIP (Schlaberg et al., 2014; Hamdi et al., 2020; Wang et al., 2022; Yang J. et al., 2023). For *N. brasiliensis*, we showed 54.6% the sensitivity rate to CRO, and 0% sensitive to IPM, and other studies showed relatively low sensitivity rates to CRO (among 2% - 49%) and IPM (only 0% - 8%) (Schlaberg et al., 2014; Hamdi et al., 2020; Wang et al., 2022; Yang J. et al., 2023; Han et al., 2024). These differences may be related to the presence of the *mtrA* gene, which encodes multi-drug resistance efflux pump transcription activator, in *N. otitidiscaviarum* and *N. brasiliensis* in our study (Valdezate et al., 2017; Lebeaux et al., 2019). Overall, *N. otitidiscaviarum* is intrinsically resistant to β-lactam antibiotics. Apart from *N. farcinica*, other species had higher sensitivity to CRO than to IPM.

Additionally, AMK and TOB separately showed 98.3% and 95% drug susceptibility to *N. cyriacigeorgica*, but TOB showed the lower sensitivity rates (56.3%) to *N. otitidiscaviarum*, which also differed from the previously published resistance patterns in the CLSI standard M24S-Ed2. However, we had not found the resistance genes related to aminoglycoside antibiotics, it may be related to virulent factors such as *sigA/rpoV*, *pafA* and *groEL2* genes, ubiquitously present in these strains. In addition, our study found that 32.2% of *Nocardia* isolates were resistant to three or more commonly used antibiotics, indicating the widespread presence of MDR. Valdezate S reported the MDR varied from 1% in *N. cyriacigeorgica* and *N.* abscessus to 10% in *N. brasiliensis*, 20% in *N. transvalensis*, 35% *N. farcinica* and *N. otitidiscaviarum* (Valdezate et al., 2017). But Song Z reported 38.5% of *Nocardia* isolates were resistant to two or more commonly used antibiotics (AMK, CRO, IPM and TMP-SMX) as MDR, with 29.6% for *N. farcinica*, 45% for *N. cyriacigeorgica*, and 100% for *N. otitidiscaviarum* (Song et al., 2025).

Through analysis of resistance genes and virulence genes, we observed a strong correlation between genotype and phenotype. It is reasonable to assume that the differences in the resistance phenotypes of *Nocardia* among different countries or regions are not only related to the isolated areas and the strains number included in the study, also suggest that the differences in resistance and virulence of *Nocardia* isolates between studies, which in turn may account for the different outcomes (Yetmar et al., 2023b), and meaning that the resistance transmission mechanisms of *Nocardia* are worthy of further attention. These findings provide key insights into the different resistance patterns and genotypes of *Nocardia* strains, thereby emphasizing the importance of continuous genomic surveillance and personalized treatment for different nocardiosis. Nevertheless, the resistance observed in some strains lacking known resistance determinants indicates the presence of uncharacterized mechanisms that require further investigation.

Nocardiosis usually requires long - term treatment, which is often associated with significant toxicity and drug - drug interactions. Moreover, most medical institutions have difficulties in obtaining AST results, which means that the antibiotic management of nocardiosis requires clinicians to choose empirical treatment based on the severity of the infection and local epidemiology (Besteiro et al., 2023). In cases of non - severe pulmonary disease or primary cutaneous nocardiosis, TMP-SMX monotherapy is recommended by many authors (Restrepo and Clark, 2019; Margalit et al., 2021). In severe disease or CNS infection, combination therapy with at least two drugs is recommended to ensure that at least one drug is effective, or LZD monotherapy is also a good choice, and the initial multi-drug regimen usually includes IPM, AMK and TMP-SMX (Restrepo and Clark, 2019). In our study, for simple pulmonary nocardiosis, about 96% (24 of 25) received TMP-SMX monotherapy with a success rate of 91.7% (22 of 24). About 92.1% (81 of 88) received combination therapy based on TMP-SMX with a success rate of 80.3% (65 of 81). For simple cutaneous and soft - tissue nocardiosis, the success rate was 100%, regardless of TMP-SMX monotherapy, combination therapy based on TMP-SMX or debridement used. In cases of pulmonary nocardiosis combined with two or multiple-site infections, even after aggressive clinical treatment, 26.7% failure of patients still experienced, which might be due to the severity and complexity of the patients’ underlying diseases.

But intolerance to TMP-SMX will pose a big challenge to treatment. A case of *N. farcinica* causing brain abscess had shown that alternative regimen -IPM, AMK, AMC and MNO - combination could achieve sustained remission. Individualized treatment based on AST and patient factors is crucial (Hong et al., 2025). Saksena R reported two cases of fatal pulmonary infections caused by *N. otitidiscaviarum* in elderly patients, both of whom were empirically treated with TMP-SMX but died on the 2th and 5th days after admission, respectively. However, their AST showed resistance to TMP-SMX, AMC, and IPM (Saksena et al., 2020). We support this conclusion that pulmonary nocardiosis, cutaneous and soft-tissue infections, and treatment with TMP-SMX are independently associated with 90-day all-cause mortality (Peleg et al., 2007).

Our study is limited by several factors. First, the lack of representation of all species within the *Nocardia* genus, and the number of some *Nocardia* species were insufficient, thus their results were not representative. Second, the majority of the isolates were concentrated in several cities in Henan Province, therefore, the observed disparity in strains distribution were more likely attributable to submission bias, so it could not reflect the true regional epidemiological characteristics. Correspondingly, a wider-range surveillance program should be further developed. Third, the clinical outcome analysis was purely descriptive, accordingly, no causal conclusions regarding treatment efficacy could be drawn. Finally, WGS had been performed only on some strains whose resistance patterns were inconsistent with known resistance profiles. It is necessary to expand the number of strains to detect the prevalence of the resistance determinants of *Nocardia* as a whole or individual species and analysis their correlation with resistance phenotypes, meanwhile, the bioinformatic analysis will be needed to extensively illustrate phylogeny and determination of antimicrobial resistance genes and mutations.

In conclusion, the present study is multicenter retrospective study with 214 *Nocardia* strains covering 9 years and 9 different public hospitals in Henan Province. We reported not only the clinical features and epidemiological characteristics of nocardiosis but also the most frequent and susceptibility patterns of *Nocardia* species. We also elaborated the susceptibility patterns of clinical therapeutic drugs used for this infection. These findings suggest the need to understand the *Nocardia* microbiology better so that clinical treatment methods can be optimized to prevent unfavorable outcomes. Moreover, the looming threat posed by *MDR Nocardia* isolates and resistance mechanism, as well as the potential for resistance transmission should be noted, which will help to identify the key drug targets for combating MDR *Nocardia* in the future. Nevertheless, the AST methods must be standardized. Methodological challenges (e.g., preparation and treatment of bacterial suspensions, inoculum consistency, and interpretation of cutoffs) and growth characteristics of *Nocardia* appear to be responsible for limited reproducibility of broth microdilution, especially for some drugorganism combinations (Schlaberg et al., 2014). Likewise, AST *in vitro* should provide to clinical patients as much as possible, contributing an effective basis for the clinical management of nocardiosis.

## Data Availability

The datasets presented in this study can be found in online repositories (Accession number: SUB15978095). The names of the repository/repositories and accession number(s) can be found in the article/**Supplementary Material**.
