# Supplementary material for: Epidemiology, clinical treatment and outcomes, susceptibility patterns and genotypic analysis of 214 Nocardia strains from multiple centers in Henan Province
Source: Front Cell Infect Microbiol. 2026 Feb 13;16:1728269. doi: 10.3389/fcimb.2026.1728269 (PMC12945825; doi:10.3389/fcimb.2026.1728269)
Supplement: Supplementary file 1 [file SupplementaryFile1.docx]

**Table S1 Comparison of intrageneric identification accuracy between MALDI-TOF MS and 16S rRNA sequencing for 55 *Nocardia* isolates.**

|  |  |  |  |  |
| --- | --- | --- | --- | --- |
| **Strains (16S rRNA sequencing )** | **No.** | **Identification of MALDI TOF MS (VITEK MS, IVD, version 3.0)^*^** | **Score** | **The identification accuracy rate within the genus** |
| *N. abscessus* | 11 | 3 for *N. beijingensis* and 8 for *Nocardia sp.* | 65.0-90.0 | 94.5% |
| *N. beijingensis* | 7 | 1 for *N. abscessus, 2 for N. asteroides* and 5 for *Nocardia sp.* | 70.0-95.0 |  |
| *N. nova* | 5 | 4 for *N. nova/africana*, and 1 for *Nocardia sp.* | 88.0-95.0 |  |
| *N. puris* | 1 | *N. abscessus* | 85.0 |  |
| *N. asiatica* | 5 | 1 for *N. abscessus*, 1 for *N. beijingensis*, 2 for *N. asteroides* and 1 for *Nocardia sp.* | 60.0-88.0 |  |
| *N. carnea* | 1 | *Nocardia sp.* | 80.0 |  |
| *N. vulneris* | 1 | *Nocardia sp.* | 80.0 |  |
| *N. yamanashiensis* | 1 | *Nocardia sp.* | 78.0 |  |
| *N. amamiensis* | 12 | 5 for *N. beijingensis,* 1 for *N. asteroides* and 6 for *Nocardia sp.* | 65.5-95.0 |  |
| *N. pneumoniae* | 1 | *Nocardia sp.* | 95.0 |  |
| *N. bhagyanarayanae* | 1 | *Nocardia sp.* | 80.0 |  |
| *N. cyriacigeorgica* | 1 | *Nocardia sp.* | 80.0 |  |
| *N. otitidiscaviarum* | 1 | *Nocardia sp.* | 75.5 |  |
| *N. niwae* | 2 | *Nocardia sp.* | 70.0 |  |
| *N. coubleae* | 1 | *N. paucivorans* | 80.0 |  |
| *N. farcinica* | 1 | *Nocardia sp.* | 75.0 |  |
| *non-Nocardia^**^* | 3 | 2 for *Micromonospora chokoriensis* and 1 for *Saccharopolyspora hordei* | 70.5-88.5 |  |
|  |  |  |  |  |

^*^ Identification method: formic acid extraction was used, and 1 µL of the extract was spotted in triplicate onto the MALDI-TOF MS target plate. The procedure was the same as that employed for all *Nocardia* species.

^**^ For the 3 misidentified isolates, their smear morphology and culture characteristics can easily be confused with those of certain *Nocardia* species. The mainly manifesting as: the hypha were delicate and thin; the growth of colonies were extremely slow which remained tiny, and gray-white even after incubating for 5-10 days on blood agar, and showed the “agar-biting” phenotype; moreover, weak acid-fast staining results were variable.

**Table S2 Antibiotic resistance genes in the 7 species of *Nocardia spp.* genome according to the CARD**

|  |  |  |  |  |
| --- | --- | --- | --- | --- |
| **Resistance gene type** | **Gene name** | **Gene ID** | **Description** | **Resistance Mechanism** |
| β-lactam antibiotics | FAR-1 | ARO:3004784 | A class A beta-lactamase gene found in N. farcinica. | antibiotic inactivation |
| macrolide and lincosamide antibiotics | ErmB | ARO:3000375 | encoding a S-adenosylmethionine-dependent 23S rRNA adenine N6-methyltransferase, which can alter the conformation of the bacterial ribosome, thereby conferring resistance to these antibiotics. | ribosomal modification inducible expression |
| β-lactam antibiotics | AST-1 | ARO:3004740 | Broad-spectrum beta-lactamase gene | antibiotic inactivation |
| sulfonamide antibiotics | sul1 | ARO:3000410 | encoding a variant of dihydropteroate synthase (DHPS), an enzyme that can resist the inhibitory effects of sulfonamide drugs. The sul1 gene is typically located within composite transposons, such as the IS6100 composite transposon. These transposons can act as vectors for the spread of resistance, mediating the horizontal transfer of resistance genes between *Nocardia* species or with other genera. | encoding dihydropteroate synthase (DHPS) transposon-mediated dissemination |
| Glycopeptide antibiotics | vanRO | ARO:3002930 | Vancomycin resistance operon genes, which can synthesize peptidoglycan with modified  C-terminal D-Ala-D-Ala to D-alanine-D-lactate | transcriptional activation |
| a variety of antibiotics,including β-lactam antibiotics, rifampicin, macrolides, etc. | mtrA | ARO:3000816 | encoding a transcriptional activator of a multidrug efflux pump. | [antibiotic efflux](https://card.mcmaster.ca/ontology/36001) |
| Tetracycline antibiotics | tetA_60 | ARO:3004035 | Tetracycline efflux pump | [antibiotic efflux](https://card.mcmaster.ca/ontology/36001) |
| Tetracycline antibiotics | tetB_60 | ARO:3004036 | Tetracycline efflux pump | [antibiotic efflux](https://card.mcmaster.ca/ontology/36001) |
| Tetracycline antibiotics | tetM | ARO:3000186 | Tetracycline efflux pump | [antibiotic efflux](https://card.mcmaster.ca/ontology/36001) |
| rifamycin antibiotic | rpoB2 | ARO:3000501 | Expression of the rpoB2 variant results in replacement of rifampin sensitivity with rifampin resistance | antibiotic target alteration, antibiotic target replacement |
| Glycopeptide antibiotics | vanR_in_vanO_cl | ARO:3002930 | Vancomycin resistance operon genes, which can synthesize peptidoglycan with modified  C-terminal D-Ala-D-Ala to D-alanine-D-lactate | [antibiotic efflux](https://card.mcmaster.ca/ontology/36001) |
| rifamycin antibiotic | Nfar_rox | ARO:3007210 | Inactivates rifampicin by encoding a rifampicin monooxygenase. | antibiotic inactivation |
|  |  |  |  |  |

**Table S3 Virulence gene candidates in the 7 species of *Nocardia spp.* according to the VFDB**

| **Category** | **Gene name** | **Gene ID** | **Description** |
| --- | --- | --- | --- |
| Adherence | plr/gapA | VFG005359 | type I glyceraldehyde-3-phosphate dehydrogenase |
|  | groEL2 | VFG043550 | molecular chaperone GroEL |
| Regulation | sigA/rpoV | VFG009718 | RNA polymerase sigma factor |
|  | sigE | VFG009749 | RNA polymerase sigma factor |
|  | regX3 | VFG031730 | two-component sensory transduction protein RegX |
| Stress survival | pafA | VFG031093 | Pup-protein ligase\|Proteasome associated proteins |
|  | pafA | VFG031092 | Pup-protein ligase\|Proteasome associated proteins |
|  | pafA | VFG031101 | hypothetical protein |
|  | mpa | VFG031184 | putative ATPase |
|  | mpa | VFG031190 | proteasome ATPase |
|  | ahpC | VFG009680 | peroxiredoxin |
|  | ahpC | VFG022829 | putative alkylhydroperoxidase C |
| Immune modulation | MYCSM RS30910 | VFG029754 | YciI family protein |
|  | rfbA | VFG006052 | glucose-1-phosphate thymidylyltransferase RfbA |
|  | rfbB | VFG006022 | dTDP-glucose 4,6-dehydratase |
|  | rfbD | VFG006081 | dTDP-4-dehydrorhamnose reductase |
|  | STR_RS06885 | VFG006057 | alpha-L-Rha alpha-1,3-L-rhamnosyltransferase |
|  | STR_RS06895 | VFG006065 | ABC transporter ATP-binding protein |
|  | STER_RS07065 | VFG006067 | glycosyltransferase family 2 protein |
|  | STR_RS06900 | VFG006069 | ABC transporter permease |
|  | STR_RS06910 | VFG006077 | glycosyltransferase family 1 protein |
|  | STU_RS16245 | VFG006084 | ABC transporter permease |
|  | STU_RS16250 | VFG006086 | glycosyltransferase family 2 protein |
|  | STU_RS16255 | VFG006088 | glycosyltransferase family 1 protein |
|  | STU_RS16260 | VFG006090 | glycosyltransferase family 2 protein |
|  | STU_RS16265 | VFG006091 | lipopolysaccharide biosynthesis protein |
